# A Variable Stiffness Actuator Module With Favorable Mass Distribution for a Bio-inspired Biped Robot

**DOI:** 10.3389/fnbot.2019.00020

**Published:** 2019-05-17

**Authors:** David Rodriguez-Cianca, Maarten Weckx, Rene Jimenez-Fabian, Diego Torricelli, Jose Gonzalez-Vargas, M.Carmen Sanchez-Villamañan, Massimo Sartori, Karsten Berns, Bram Vanderborght, J. Luis Pons, Dirk Lefeber

**Affiliations:** ^1^Robotics and Multibody Mechanics Research Group, Vrije Universiteit Brussel (VUB) and Flanders Make, Brussels, Belgium; ^2^Cajal Institute, Spanish National Research Council (CSIC), Madrid, Spain; ^3^Ottobock GmbH, Duderstadt, Germany; ^4^Department of Biomechanical Engineering, University of Twente, Enschede, Netherlands; ^5^Robotics Research Lab, University Kaiserslautern, Kaiserlslautern, Germany

**Keywords:** variable stiffness actuator, bio-inspired biped robot, mass distribution, muti-DoFs joints, human-like locomotion

## Abstract

Achieving human-like locomotion with humanoid platforms often requires the use of variable stiffness actuators (VSAs) in multi-degree-of-freedom robotic joints. VSAs possess 2 motors for the control of both stiffness and equilibrium position. Hence, they add mass and mechanical complexity to the design of humanoids. Mass distribution of the legs is an important design parameter, because it can have detrimental effects on the cost of transport. This work presents a novel VSA module, designed to be implemented in a bio-inspired humanoid robot, Binocchio, that houses all components on the same side of the actuated joint. This feature allowed to place the actuator's mass to more proximal locations with respect to the actuated joint instead of concentrating it at the joint level, creating a more favorable mass distribution in the humanoid. Besides, it also facilitated it's usage in joints with centralized multi-degree of freedom (DoF) joints instead of cascading single DoF modules. The design of the VSA module is presented, including it's integration in the multi-DoFs joints of Binocchio. Experiments validated the static characteristics of the VSA module to accurately estimate the output torque and stiffness. The dynamic responses of the driving and stiffening mechanisms are shown. Finally, experiments show the ability of the actuation system to replicate the envisioned human-like kinematic, torque and stiffness profiles for Binocchio.

## 1. Introduction

Creating bipedal robots that can walk stably and efficiently as humans has been an open challenge since long time in robotics research (Vukobratović and Borovac, 2004). Traditional approaches focus on multiple degree-of-freedom (DoF) platforms controlled by classic control paradigms that ensure quasi-static stability, e.g., the Zero-Moment Point (ZMP) (Vukobratović, 1975; Vukobratović and Borovac, 2004). Despite their good performance on flat terrain, most of these robots show important limitations, such as high energetic costs, slow walking motion, poor robustness on uneven terrains, and unnatural kinematic patterns (Torricelli et al., 2016). Different from this approach, the “dynamic walking” principle emerged to improve the human-like properties of bipeds, realizing natural, and efficient motion with little or no actuation (McGeer, 1990; Collins et al., 2005; Hobbelen and Wisse, 2005). These solutions, while minimizing kinematic and control complexity, show poor stability, versatility and controllability in realistic environments. Compliant actuation has been proposed to narrow the gap between these two approaches. In humans, the intrinsic compliant properties of joints and muscles are at the basis of robust, energy efficient, and versatile locomotion. Humans modulate the stiffness of the joints through the co-contraction of agonist and antagonist muscles, producing large ranges from rigid to highly compliant behaviors (Sartori et al., 2015). These mechanisms are central for adapting to a large variety of terrains (Ferris et al., 1998) and for naturally adjusting to biomechanical and energetic demands (Farley and Gonzalez, 1996).

Series Elastic Actuators (SEAs) introduce compliance to humanoid robotics, resulting in safer human-robot interaction, shock absorption, and greater energy efficiency compared to stiff actuators (Vanderborght, B. et al., 2013). However, modulation of joint stiffness can only be achieved through impedance control of the SEAs (Kim et al., 2012; Tsagarakis et al., 2013; Paine et al., 2014; Pierce and Cheng, 2014; Negrello et al., 2016). Variable Stiffness Acuators (VSAs), on the other hand, can inherently change the mechanical compliance of a humanoid's joints, having positive outcomes in terms of energy efficiency, robustness against disturbances and similarity with human motions due to their inherent compliant behavior. Previous research regarding the application of VSAs in humanoids showed modulation of walking velocity and step length in passive dynamic walkers (Huang et al., 2013), and the postural control of a biped (Hettich et al., 2011). Currently, only few examples of bipeds with VSAs can be found: Veronica (Huang et al., 2013), Lucy (Vanderborght et al., 2008), and BLUE/miniBLUE (Enoch and Vijayakumar, 2015). Several working principles for actuators with variable-stiffness capabilities have been proposed for various robotic applications. Examples of these are the Compliant Asymmetric Antagonistic Actuator (Roozing et al., 2015), vsaUT-II (Groothuis et al., 2014), AwAS-II (Jafari et al., 2012), Variable Torsion Stiffness Actuator Schuy et al. (2013), Mechanically Adjustable Compliance and Controllable Equilibrium Position Actuator (MACCEPA) (Van Ham et al., 2007), and ARES (Cestari et al., 2015). Because of the importance of multi-DoF joints in the human body, bio-inspired robotic applications often require multi-DoF actuated joints (Mizuuchi et al., 2007; Potkonjak et al., 2011; Nakanishi et al., 2012). In these applications, nonetheless, no variable stiffness capabilities were implemented. Instead, the compliance of such actuators was fixed. The research on multi-DoF actuators with variable stiffness is, therefore, still limited. Generally, cascades of single-degree of freedom actuators are used (Catalano et al., 2011). One of the few examples that follows a more integrated approach is the multi-DoF actuator with variable stiffness based on two antagonistic setups of ANLES actuators (Koganezawa et al., 2012). Another example, based on the MACCEPA concept, is proposed in Weckx et al. (2014). VSAs bring about increased mechanical complexity and weight since generally two motors are required for the independent control of both equilibrium position and stiffness. Mass distribution of the legs is an important design parameter, because it can have detrimental effects on the cost of transport. It is reported that the net metabolic cost of walking increases with more distal location of increased load mass. Hence increased load mass at the foot has greater effect on the metabolic cost than at the thigh (Browning et al., 2007; Schertzer and Riemer, 2014). Added mass to lower extremities also increases the swing leg's moment of inertia about the hip joint, resulting in higher moments at the knee and ankle compared to normal walking (Royer and Martin, 2005). This in turn leads to heavier motors in a humanoid's legs. Mass and the distribution of mass are therefore key aspects in the design of humanoids.

This work presents a novel VSA module designed to be implemented in the sagittal DoFs of the legs of Binocchio, a bio-inspired humanoid robot designed as platform for the validation of biomimetic controllers and the understanding of the neuromechanical processes of human movement, including the role of compliance during walking. The presented VSA module places both motors, the driving and the stiffening motor, on the same side of the actuated joint, previously not possible with existing concepts, and in-line with the housing link. This feature allows to place the actuator's mass to more proximal locations with respect to the actuated joint instead of concentrating it at the joint level, creating a more favorable mass distribution in the design of the humanoid's leg. Besides, it also facilitates it's usage in joints with centralized multi-DoFs joints instead of cascading single DoF modules. These innovations have been illustrated with the implementation of the VSA module in the compliant multi-DoF joints of the humanoid biped Binocchio. The requirements for the VSA module, it's overall working principle, mechanical design and first prototype are presented in section 2. Section 2.5 shows the integration of the proposed actuator in the multi-DoFs joints of Binocchio. Subsequently, the static characteristics of the VSA module, the dynamic responses of the driving and stiffness modulation mechanisms and the ability of the actuator to follow human-like kinematics, torque and stiffness profiles are experimentally validated in section 3. Discussion of the presented results and conclusions from this work are given in sections 4 and 5.

## 2. A Novel VSA Module for a Bio-Inspired Humanoid Robot

### 2.1. Requirements

**Figure 1:**
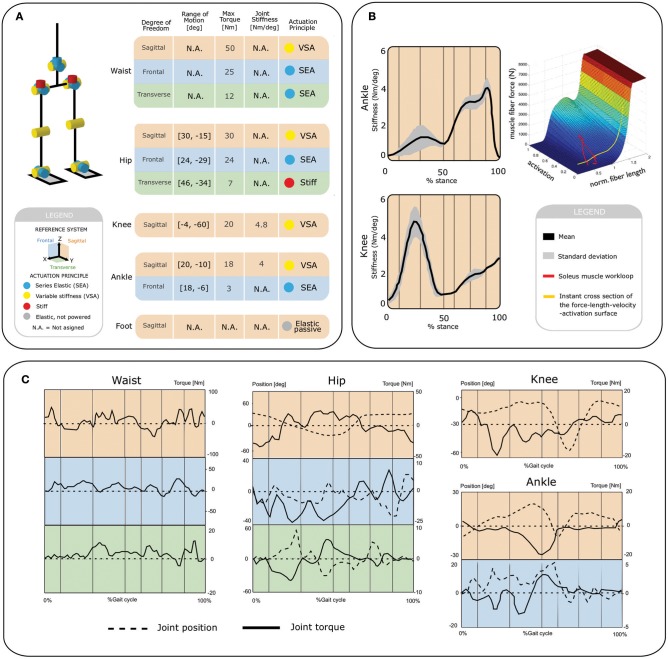
Actuation requirements for Binocchio. **(A)** Kinematic and actuation concept, **(B)** Estimates of human stiffness modulation for the human ankle and knee sagittal joints. **(C)** Kinematic and actuation profiles derived from the human-like B4LC simulator.

Figure 1A shows the defined joint actuation scheme for the Binocchio biped, based on a study of the key principles of human locomotion with a special focus on the relevance of lower limb joints' compliance during walking (Torricelli et al., 2016). The actuator presented in this paper is aimed at being implemented in all the biped's sagittal joints, i.e., waist, hip, knee and ankle as, based on the previous work, variable stiffness seems to play a bigger role in these DoFs during ground-level human locomotion. We therefore used modeling and simulation approaches in combination with evidence from human studies in order to define the actuation requirements. We employed a simulated biomimetic biped, called B4LC (Luksch and Berns, 2010), i.e., Bio-inspired Behavior-Based Bipedal Locomotion Control, to generate realistic estimates of human-like kinematic and torque patterns based on the biped's weight (35 kg) and height (170 cm) for level ground walking at a cadence of 1.4 s/stride (Figure 1C). As all the VSA actuators were meant to have the same specifications, we based the requirements on the maximum values observed in the simulations. We defined a maximum required range of motion (ROM) of 90° and a maximum torque of 40 Nm. As for the requirements on the joint stiffness modulation, we used a model, developed by Sartori et al. (2015), to predict stiffness changes in the joints throughout the gait cycle for the knee and ankle joints (Figure 1B). Based on this model we defined a required stiffness range between 0 and 5 Nm/deg. As for the hip joint, we defined the same stiffness range as in agreement with the results presented in Shamaei et al. (2013). Since no evidence was available on the stiffness variation in the waist, the same stiffness range was defined.

### 2.2. Conceptual Development

**Figure 2:**
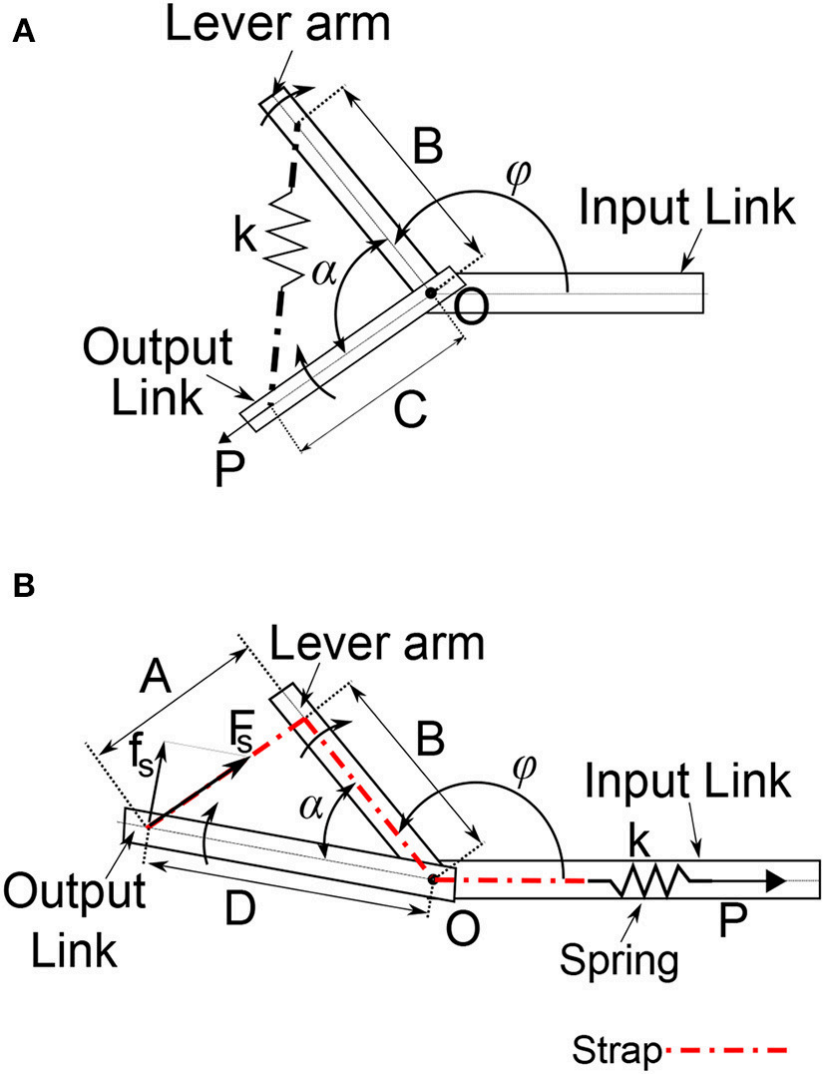
Schematics of the MACCEPA: **(A)** conventional design showing the elastic element spanning the actuated joint and **(B)** novel concept showing the elastic element housed in the reference body (Link 1).

The VSA actuator presented in this work is based on the MACCEPA concept (Van Ham et al., 2007). In the original concept (Figure 2A), the spring is housed on the output link, which means both the mechanisms used to drive the lever arm and the one to change the pre-compression on the spring, *P*, are housed on different links of the joint (namely input and output link, respectively). In contrast, the novel MACCEPA concept presented in this paper (Figure 2B) allows both mechanisms to be housed in the input link. This represents an advantage since the actuator's mass can be placed in more proximal locations to reduce the inertia of the output link. Besides, since the actuator's components are housed on the same link of the joint (the input link), the remaining link (the output link) can house a second actuator to drive a second DoF by means of a universal joint, where each of the actuators drive one of the axis of the 2 DoFs joint. A strap is attached to the output link (OL) at a distance *D* from the axis of rotation, denoted by *O* in the diagram. The strap is subsequently guided through the lever arm (LA). The strap is guided through the joint's axis of rotation *O* to a linear spring housed on the input link, to which it is rigidly attached. The effective length of LA, *B*, is defined as the distance between the axis of rotation and the point where the strap makes initial contact with LA. When LA and OL are aligned, the force exerted by the strap, due to the initial deformation of the spring, or initial pre-compression, is aligned and no torque is exterted on OL. When α is different from zero, an additional deformation is added to the strap, which exerts a force on the spring *F*_S_. The exerted force is no longer aligned with OL, resulting in a torque determined by the perpendicular component of *F*_S_ with respect to the OL at a distance *D* from the joint's center of rotation. By changing the initial pre-compression force *P* in the spring, the actuator's output stiffness can be adjusted. One of the practical features of the proposed design is that the actuator's output torque and stiffness characteristics (or quasi-stiffness Rouse, E.J. et al., 2013) can be easily and accurately predicted using only the deviation angle α, and the force in the strap, *F*_S_(*t*), or the spring linear deformation, *p*(*t*), induced by the compression, or stiffening mechanism. The torque-angle relationship is given by

(1) $$\begin{array}{l} T=T(\alpha ,p)=D\cdot f_{S}\\ \;=k\left(A(\alpha )+B+p(t)-D\right)\frac{BD}{A(\alpha )}\text{sin}(\alpha ) \end{array}$$

or

(2) $$T=T(\alpha ,F_{S})=F_{S}(t)\frac{BD}{A(\alpha )}\text{sin}(\alpha )$$

where *F*_S_(*t*) is the force in the strap and

(3) $$A(\alpha )=\sqrt{B^{2}+D^{2}-2BD\text{ cos}(\alpha )}$$

is the length between the attachment points of the strap at the output link and the lever arm. *T* = *T*(α, *F*_S_) can be used to improve the torque prediction if *F*_S_ is directly measured, which is convenient in situations when the spring stiffness *k* is unknown or non-linear. The force in the strap and the deformation of the spring induced by the compression mechanism are related through

(4) $$p(t)=\frac{1}{k}F_{S}(t)-A(\alpha )-B+D.$$

When *p*(*t*) = *p* is held constant, the force in the spring due to it's initial deformation is *P* = *k*·*p* = *F*_S_(0) when the actuator is at equilibrium position (α = 0).

This relatively simple representation, given by (1)–(4), can effectively be used to estimate the actual output torque for design purposes as well as in closed-loop feedback schemes. The partial derivative of (1) with respect to α yields the output stiffness *S* of the actuator (or quasi-stiffness) as a function of α for a given initial spring deformation *p*:

(5) $$\begin{array}{l} S=S(\alpha ,p)=\frac{dT(\alpha ,p)}{d\alpha }\\ \;=k\left(A(\alpha )+B+p(t)-D\right)\\ \;\times \frac{BD}{A(\alpha )}\left[\text{cos}(\alpha )-\left(\frac{BD{\text{ sin}}^{2}(\alpha )}{A{\left(\alpha \right)}^{2}}\right)\right]\\ \;+k{\left(\frac{BD\text{ sin}(\alpha )}{A(\alpha )}\right)}^{2} \end{array}$$

or

(6) $$\begin{array}{l} S=S(\alpha ,F_{S})=\frac{dT(\alpha ,F_{S})}{d\alpha }\\ \;=F_{S}(t)\times \frac{BD}{A(\alpha )}\left[\text{cos}(\alpha )-\left(\frac{BD{\text{ sin}}^{2}(\alpha )}{A{\left(\alpha \right)}^{2}}\right)\right]\\ \;+k{\left(\frac{BD\text{ sin}(\alpha )}{A(\alpha )}\right)}^{2} \end{array}$$

if the force in strap is available.

When the system is at it's equilibrium position, *S* accounts for the apparent stiff reaction of it's output link to external perturbations producing a deviation angle from the equilibrium position, as in the case of a passive torsional spring.

### 2.3. Mechanical Design

**Figure 3:**
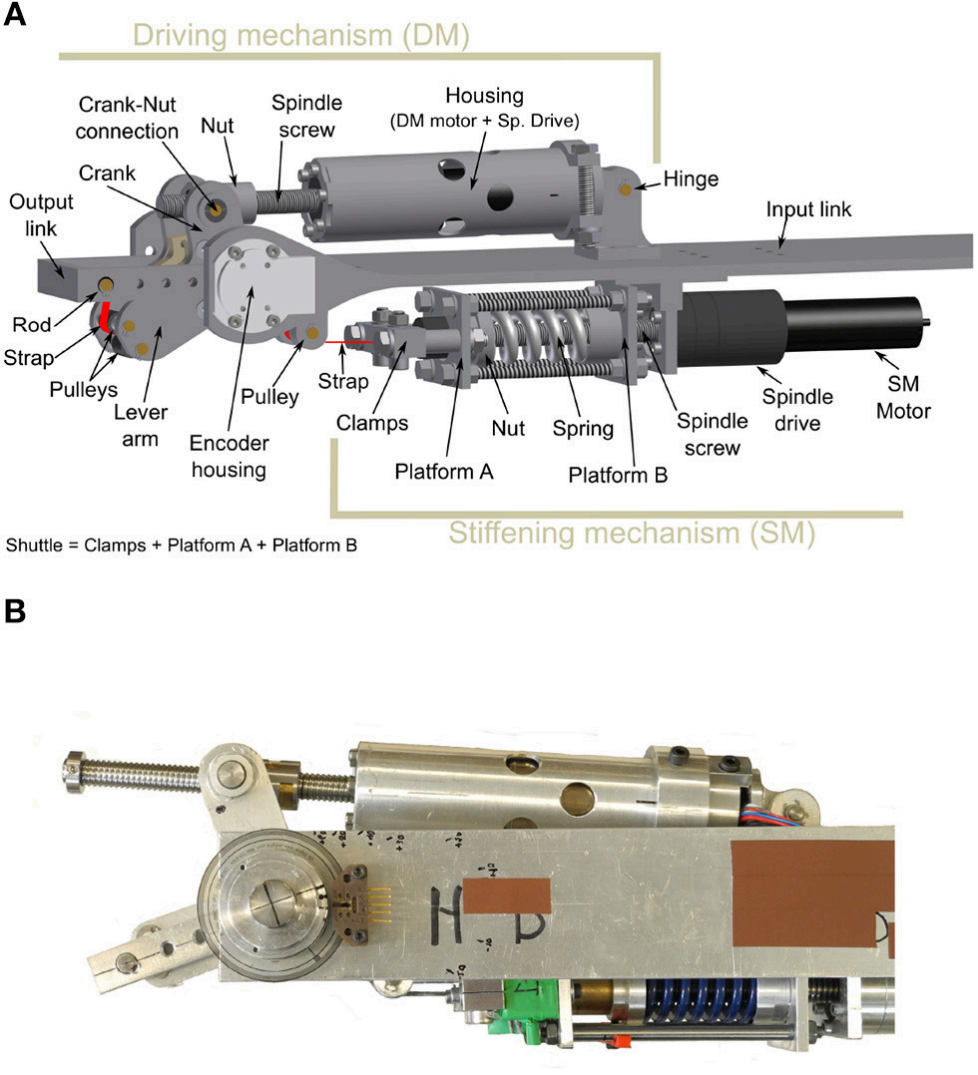
**(A)** CAD drawing of the VSA actuator design. **(B)** Prototype of the VSA.

Figure 3A shows the mechanical design of the proposed actuator based on the previously explained concept. The system consists of two mechanisms: the driving mechanism (DM) and the stiffening mechanism (SM), connected by means of a Kevlar® strap.

**Figure 4:**
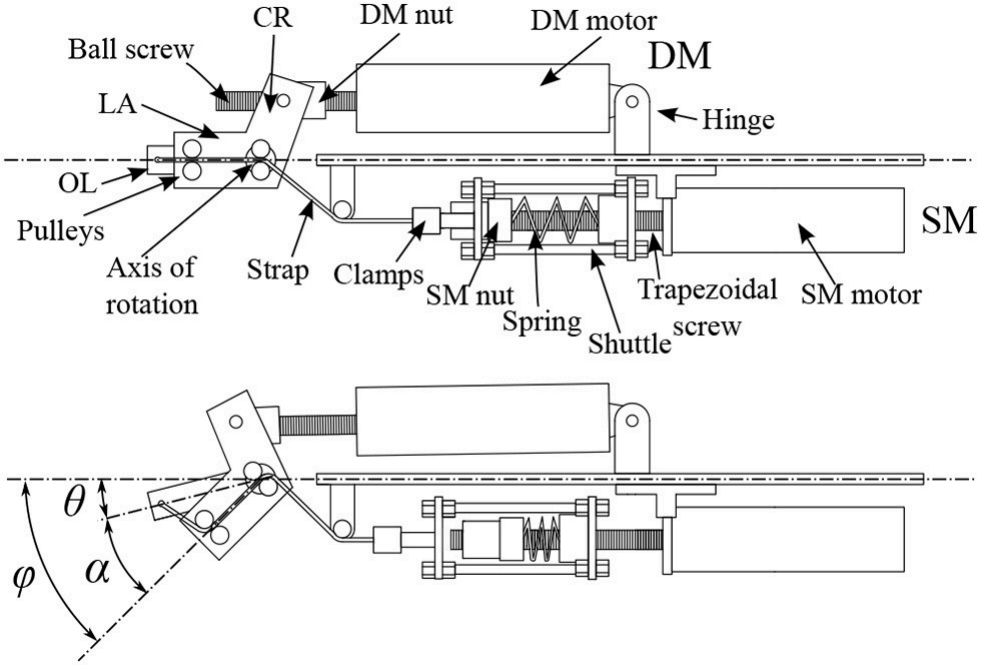
Illustration of the working principle of the actuator. In the top figure, the actuator is shown in a neutral position with no deviation angle and no pre-compression. The force exerted by the strap on the OL, due to an initial spring pre-compression, is aligned with it and no torque is produced. In the bottom figure the module is shown with a deviation angle α and an initial pre-compression. The strap pulls the shuttle over the SM nut, compressing the spring. The force exerted by the strap is no longer aligned with the OL and produces a torque around the axis of rotation that tends to re-align the LA and the OL. The pre-compression nut can be moved toward the pre-compression motor to give an initial compression to the spring and, as a consequence, modify the stiffness of the actuator.

The driving mechanism defines the position of the lever arm, and as such the position where the actuator does not generate any torque, i.e., the equilibrium position. The purpose of the stiffening, or pre-compression, mechanism is 2-fold: On the one hand, it transforms any deviation angle between the lever arm and the output link into a deformation of the spring during the operation of the actuator. On the other hand, the stiffening mechanism is able to set the initial deformation on the spring to adjust the level of compliance, or stiffness, of the output link when interacting with the environment. Figure 4 illustrates the working principle of the actuator.

#### 2.3.1. Driving Mechanism

The driving mechanism consists of a custom-made inverted slider-crank configuration that drives the lever arm (Figure 3A). A motor (EC-4pole 22, 200 W, 24 V, *Maxon motor*) in combination with a ball spindle drive (GP 32S *Maxon motor*, 1:1 transmission ∅10 × 2 mm ball) acts as the slider. The spindle's nut is connected to the crank, allowing it to rotate with respect to the nut. The crank itself is rigidly connected to the lever arm. The motor-spindle-drive combination is hinged with respect the input link so that the slider's length equals the distance between the crank-nut connection and the hinge point. The angle between the crank and the lever arm, the length of the crank, and the relative position of the hinge to the axis of rotation can be used to tune the transmission between spindle drive and the lever arm. When the spindle drive rotates, it transforms the rotational movement of the motor into a translation of the nut. When the position of the nut changes along the spindle screw, the length of the slider changes. This, in turn, changes the configuration of the inverted slider-crank and alters the angular position of LA. The linear force exerted by the spindle drive, therefore, is transformed into torque on the lever arm. A strap is fixed to the free end of the OL and enters the LA through a pair of pulleys. It exits the LA through a second pair of pulleys that guides it through the joint's axis of rotation to avoid parasitic torques. To accommodate this, the axis of rotation is not a continuous axle, but rather two axles, one on each side of the lever arm. The strap is subsequently guided toward the stiffening mechanism where it is attached to the shuttle.

#### 2.3.2. Stiffening Mechanism

The stiffening mechanism uses a carriage system to transform a deviation angle α into a deformation of the spring. A motor (EC-4pole 22, 200 W, 24 V, *Maxon motor*) directly connected in-line with a trapezoidal spindle drive (Spindle Drive GP 32 S, *Maxon motor*, 4.1:1 transmission TR ∅10 × 2 mm) is attached to the input link. The spindle's screw is centrally positioned through a carriage system. The spindle's nut is located on the far end of the motor side. As indicated in Figure 3A, the shuttle consists of a set of clamps, Platform A, and Platform B. The shuttle is centered around the spindle and is able to translate linearly relative to it. A clamp rigidly attaches the strap to the shuttle through platform A. A die spring (68.68 N/mm, *Lesjöfors*) is placed around the screw and is held in place by Platform B on one end and the spindle's nut on the other end. By changing the position of the nut with the stiffening motor while the shuttle is kept in-place, the initial compression of the spring is changed. When a force, parallel to the screw and directed away from the motor side, is applied to the shuttle, the spring compresses against the nut.

### 2.4. Prototype

Figure 3B shows the mechanical construction of the first prototype. The driving and the stiffening mechanisms are attached to different sides of an aluminum H profile. As for the materials, we employed aluminum 7,075 for most of the actuator's parts except for the joint's center of rotation, where we chose stainless steel. The DM includes two 14-bit magnetic encoders (AS5048A, *Austriamicrosystems*), to measure the deviation angle between LA and OL (α) and the angular position of the output link with respect to the input link. The shuttle accommodates a donut compression load cell (SS1108 4448N, *Toledo Transducers*) between the spring and Platform B to measure the force acting on the strap (*F*_S_) and derive the output torque. The actuator has a total weight of 1.2 kg excluding the weight of the H-profile and can generate a maximum torque of 40.0 Nm. The maximum spring compression is 21.7 mm, resulting in a maximum force in the strap of around 1, 500 N. The main configuration parameters of the actuator are summarized in Table 1. These values were the result of an optimization process to minimize the lever arm energy requirements for the specified torque trajectories.

**Table 1:** Main actuator parameters.

**Parameter**	**Nomenclature**	**Nominal value**
Strap length change	*A*(α)	–
Effective length of lever arm	*B*	46.7 mm
Effective length of fixed link	*D*	56.0mm
Spring stiffness	*k*	68.7 N/mm
Spring deformation	*p*(*t*)	–
Force in the spring	*F*_S_(*t*)	–
Spring pre-compression	*P*	–
Deviation angle	α(*t*)	–
Lever arm angle	φ(*t*)	–
Output link angle	θ(*t*)	–

### 2.5. Integration in the Two-Degree-Freedom Joints of Binocchio

**Figure 5:**
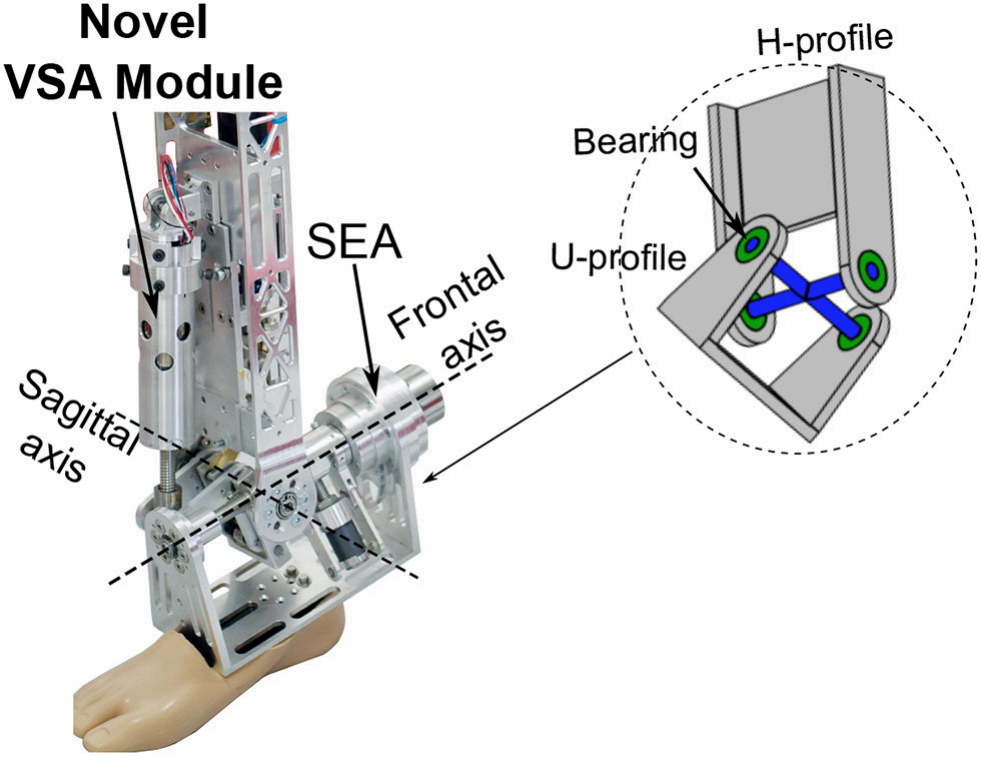
Binocchio's 2 DoFs ankle joint with a magnified view of the construction of the Cardan joint.

**Figure 6:**
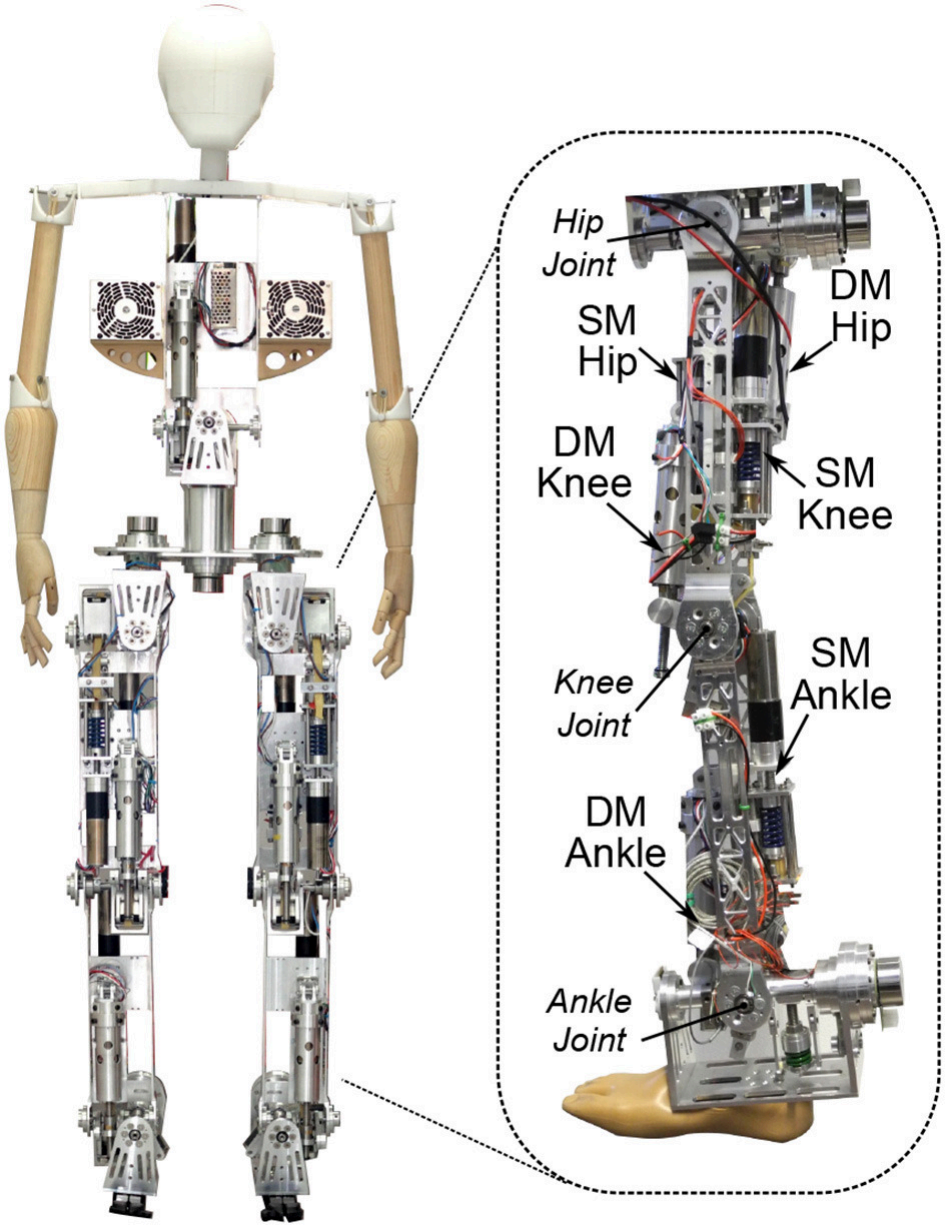
The Binocchio biped with the VSA module implemented in it's sagittal DoFs.

A common approach to construct multi-DoF joints in robotics is cascading single-DoF joints. This approach can become bulky and complex when VSAs are implemented due to the required extra motor and stiffening mechanism. A Cardan centralizes 2 DoFs in one joint and therefore is an excellent alternative to reduce the complexity and size of multi-DoFs joints. It consists of two axes rigidly and orthogonally connected to each other in the center of the joint. The 2-DoFs joints of the biped are driven by Cardan, or universal, joints. Each of the axes drives either the saggital or the frontal DoF of the joint. As shown in Figure 5, an H-shaped and a U-shaped structural profile are connected by means of bearings to the sagittal and frontal axis, respectively, of the Cardan. The VSA module is housed on the custom-made H-profile, driving the sagittal axis while a MACCEPA-based SEA module, presented in Rodríguez-Cianca et al. (2015), is housed on the U-profile, driving the frontal axis. Figure 5 shows a magnified view of the biped's 2-DoF ankle joint and an illustration showing the construction of the Cardan joint. This modular construction is used in the entire biped for the ankle, hip and trunk joints. This results in a mass-wise human-like, tapered leg with one SEA housed on the ankle, one VSA in the shank, 2 VSAs in the thigh, one SEA on the hip, and one VSA in the trunk (Figure 6).

## 3. Experiments

### 3.1. VSA Characterization

**Figure 7:**
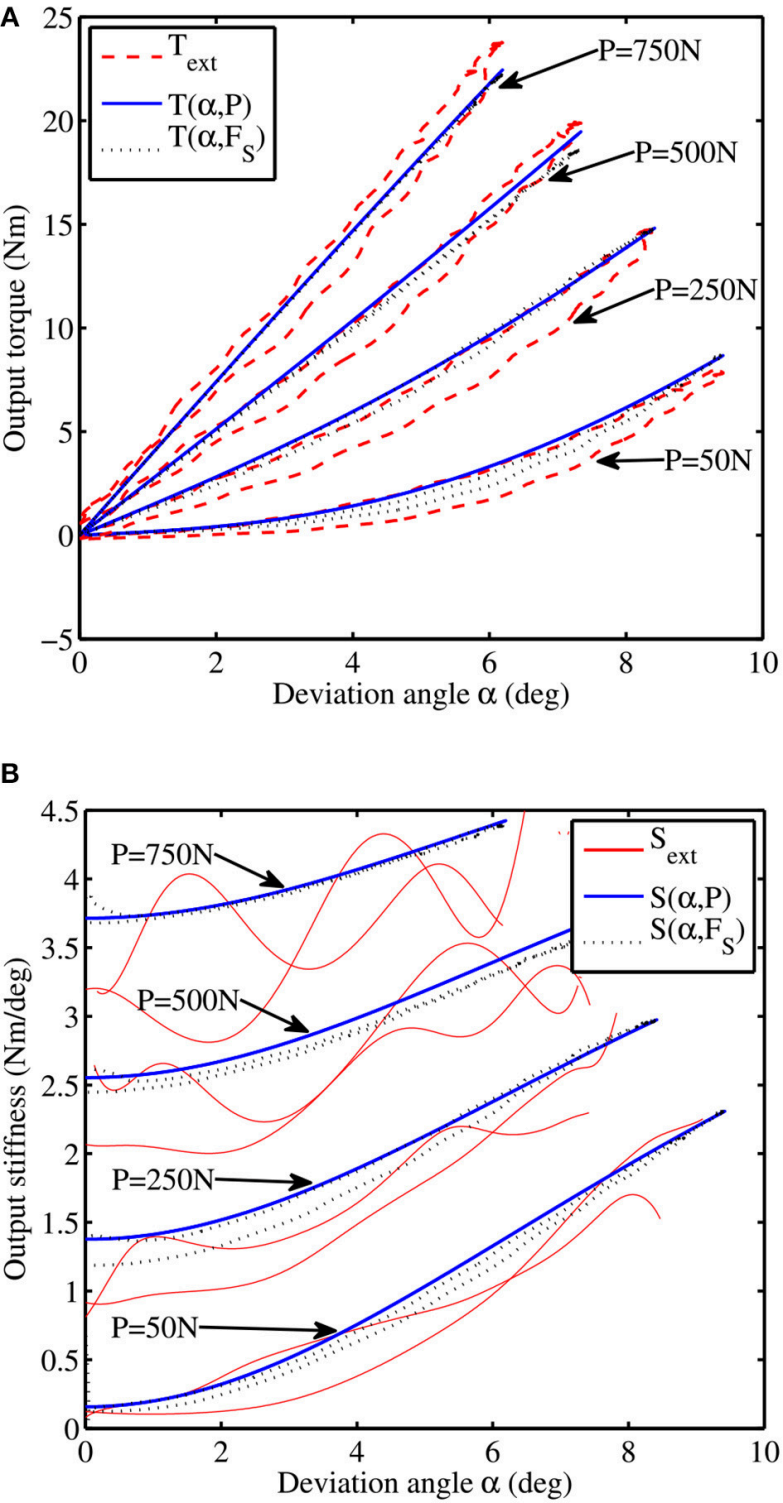
Experimentally validated characteristics of the proposed actuator: **(A)** torque-angle characteristic and **(B)** stiffness-angle characteristic.

The actuator's output torque and compliant behavior is a function of the initial spring pre-compression *P* and deflection angle α. We carried out two static tests in order to characterize this behavior and compare it with the theoretical models. We attached both the actuator and the output link to an external rigid frame, while varying the α angle by acting on the driving mechanism. We recorded the resulting torque *T*_ext_ by means of a bidirectional load cell (LSB200 445 N, *FUTEK*) placed between the output link and the external frame. We repeated the experiment for different values of initial compressions: 50, 250, 500, and 750 N, corresponding to percentages of the maximum pre-compression values of 3.3, 16.6, 33, and 50%, respectively. The readings of the internal and external load cells, as well as the lever arm encoder, where captured at a sample frequency pf 1 kHz by a real-time data-acquisition (DAQ) system (PCI-6229 *National Instruments* DAQ board on an *Intel* Core2 Duo 2.16 GHz C5102 *Beckhoff Automation* industrial computer running under *MathWorks* Real-Time Windows Target® 4.2 and Simulink® 8.1 on *Microsoft* Windows® XP). Each of the actuator's motor was driven by a commercial motor drive (ESCON 70/10 700W, *Maxon motor*) set in velocity control mode commanded by a proportional-integral- derivative (PID) force controller, in the case of the SM motor, and a proportional (P) position controller, in the case of the DM motor, with external reference inputs commanded by the DAQ system. Figure 7A shows the output torque as a function of the deflection angle α for different constant values of the initial compression force *P* in the spring using a sinusoidal angular displacement command for the lever arm with a 10° amplitude at 0.5-Hz. A comparison is made between the externally measured torque *T*_ext_ with respect to the theoretical approximations provided by (1) and (2). Using *T*(α, *p*), the root mean square error (RMSE) is 7.0%, with a maximum normalized error of 21.5%. Using *T* = *T*(α, *F*_S_), the RMSE is reduced to 6.1% with a maximum normalized error of 20.7%, possibly thanks to some dynamic effects captured by the internal load cell, errors when measuring the spring deformation or due to the fact that the spring constant *k* might not be perfectly lineal.

Figure 7B compares the output stiffness estimation using (5) and (6) for different values of the initial spring deformation. The comparison is also made against the instantaneous approximation of the derivative of *T*_ext_(*t*) with respect to α(*t*) using finite differences, denoted by *S*_ext_. This numerical approximation of *S* depicts a tendency similar to that obtained by the analytical estimation, showing a stiffening behavior as both the initial spring deformation (*P*) and deviation angle (α) values increase. The lack of accuracy of *S*_ext_, specially at higher values of *P*, could be explained by numerical errors due to the presence of noise in the input signals and possibly due to very small errors on the angle measure, which induces larger output torque estimation errors at higher pre-compression values.

### 3.2. Closed-Loop Frequency Response

#### 3.2.1. Driving Mechanism

**Figure 8:**
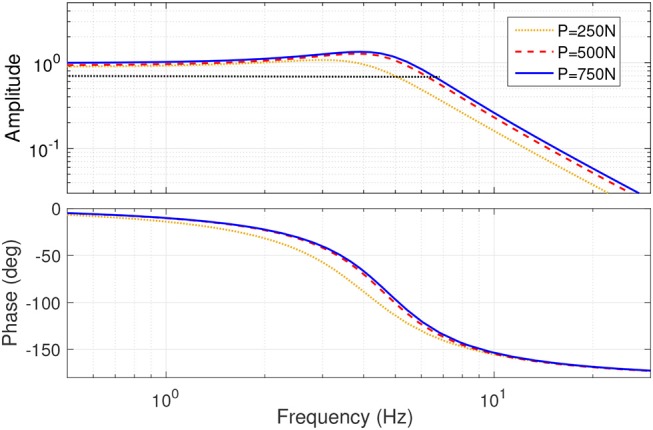
Frequency response of the driving mechanism.

The torque control of the actuator is carried out by means of the driving mechanism. Similar to former MACCEPA designs, the frequency response of this mechanism depends on the level of initial deformation applied to the actuator's spring. In order to determine this influence, we set the torque controller to follow a chirp trajectory with a peak amplitude of 10 Nm centered around 0 Nm in a frequency range between 0.05 to 10 Hz during 40 s for three different levels of initial spring compression *P* of 250 N (16% of *P*_*max*_), 500 N (33%) and 750 N (50%), while the output link was kept locked. The torque controller used the approximation *T* = *T*(α, *F*_S_) to estimate the output torque based on the position of the lever arm, α, measured by a magnetic encoder, and the force in the strap, *F*_S_, measured by a load cell placed in series with the spring. The lever arm was controlled by the DM motor, which was set in velocity control mode with a nested limiting current controller using a proportional-integral closed loop controller using the torque estimation as feedback.The system is able to exhibit different frequency-response characteristics as a function of the initial pre-compression force, as can be seen in Figure 8. The controller presented a bandwidth, calculated as the cutoff frequency at an attenuation of −3 dB, of 5.27, 6.36, and 6.53 Hz for initial pre-compression values of 250, 500, and 750 N, respectively.

#### 3.2.2. Stiffening Mechanism

**Figure 9:**
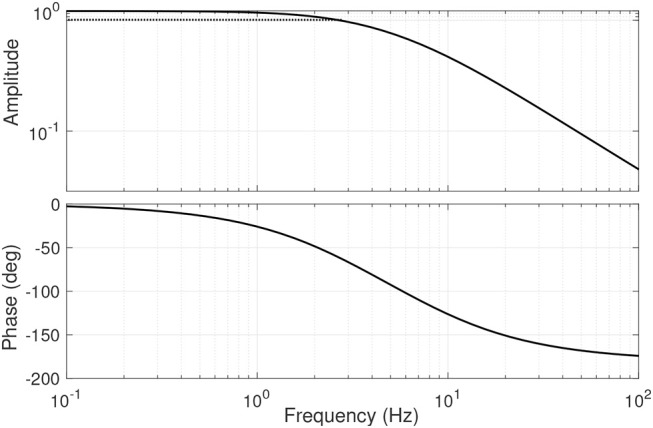
Frequency response of the stiffening mechanism.

The stiffening mechanism of the actuator is responsible of the output stiffness variation. Actuator's output stiffness is estimated using the approximation *S* = *S*(α, *F*_S_) by measuring the linear force in the strap *F*_S_ and the lever arm deviation angle α. The bandwidth of this mechanism was calculated in the absence of any external perturbance, i.e., α = 0. By modulating the force in the strap, therefore, the passive stiffness of the system can be modified. We used a simple proportional control loop to regulate the force in the Kevlar strap *F*_S_ by means of the driving mechanism motor while set in velocity control mode. The force controller was set to follow a chirp signal with a frequency swept from 0.05 Hz to 10.00 Hz in 40 s and an amplitude of 350.0 N centered around 400.0 N, corresponding to an output stiffness in the range [0.25-3.68] Nm/deg. The frequency response of the stiffening mechanism is shown in Figure 9. For the specified force command, the mechanism presents an average bandwidth of 2.23 Hz.

### 3.3. Human-Like Profiles Tracking

**Figure 10:**
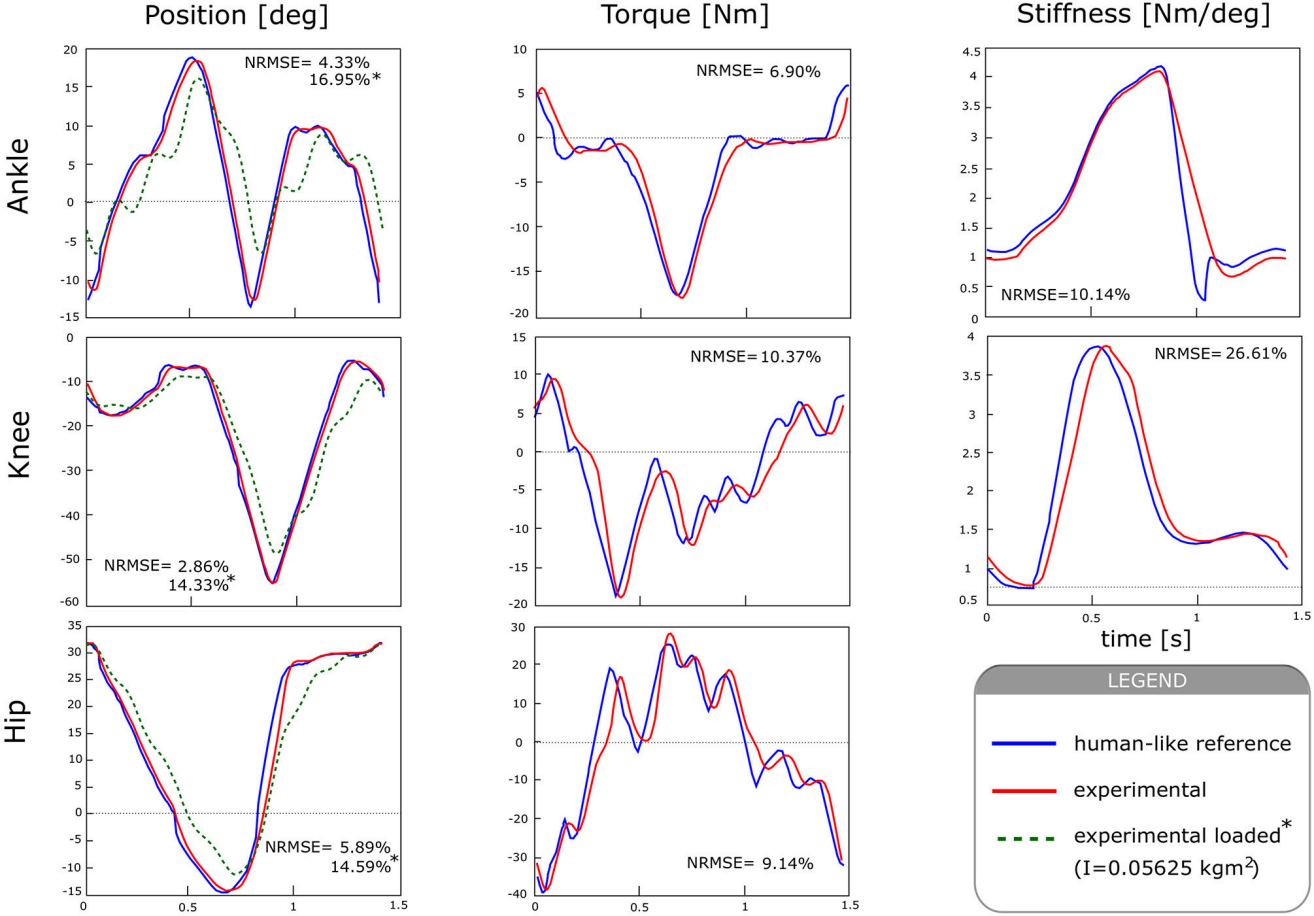
Human-like performance of the actuator, in terms of kinematics, torque, and stiffness profiles in comparison with those generated by the B4LC biped simulator.

The third set of experiments aimed at testing the ability of the VSA actuator to replicate the human-like torque, kinematics, and stiffness profiles defined in section 2.1. Results on the human-like performance of the actuator for the biped's hip, knee and ankle joints are reported in Figure 10.

To reproduce the human-like joint angle characteristics (Figure 10, left), the output link of the actuator was left free to move. The output angle was measured by means of a magnetic encoder (AS5048, AMS) with 14-bit of resolution, and used as feedback in a PID controller. In order to cancel the effect of gravity, the actuator was placed horizontally. Experiments were performed for two conditions: in the presence and in the absence of an external load, and repeated for hip, knee and ankle reference trajectories. The output load consisted of a mass of 2.5 kg attached at a distance of 15 cm from the actuator's axis of rotation, generating an inertia of 0.05625 kg m^2^. All tracking experiments were performed at a fixed pre-compression value of 200 N. PID values were manually tuned for each condition so that overshoot was limited to 5%, and data were collected for 15 consecutive cycles, which were segmented offline to get the mean and standard deviation values.

At the hip joint the actuator can follow the desired kinematic trajectory with a RMSE of 2.71 ± 0.41°, representing a normalized error of 5.89 %. In the presence of an external load, the RMSE increased to a value of 6.71 ± 0.32° (14.59 %). The knee shows an RMSE of 1.43 ± 0.45° (2.86 %) without the presence of any load, and of 7.18 ± 0.34° (14.33 %) in the presence of an external load. At the ankle joint, RMSE values were 1.41 ± 0.40° (4.33 %) and 5.51 ± 0.22° (16.96 %), without and with external load, respectively.

To test the output torque tracking performance for hip, knee and ankle joints, the generated output torque was estimated by *T* = *T*(α, *F*_S_) and used as feedback to a PI controller. The actuator could accurately follow the desired torque trajectories for the three different joints (Figure 10, center). It generated a maximum torque of 40 Nm, with peak to peak values of 65 Nm at the specified gait frequency. The maximum RMSE was 5.89 ± 0.47 Nm (9.14 %), at the hip joint. The knee and ankle joint showed an RMSE of 2.98 ± 0.24 Nm (10.37 %), and 1.62 ± 0.14 (6.90 %) respectively. These results show the actuator is able to accurately follow the desired trajectories with small tracking errors.

To generate the human-like stiffness modulation profiles (Figure 10, right), we imposed a stiffness trajectory provided by simulation (Sartori et al., 2015) on human stiffness during walking for a speed of 1.4 s/stride. The stiffness trajectory was then converted into a desired force in the strap (*F*_S_) using the approximation provided by (6) and used as setpoint for the stiffening controller. For this experiment, the angle α was kept constant at 0°. Experiments show a mean error of 1.036 ± 0.001 Nm/deg (26.61 %) for the knee and 0.424 ± 0.017 Nm/deg (10.14 %) for the ankle joint. In these experiments, the maximum required force in the spring (*F*_S_) was 790 N and 870 N for the knee and ankle, respectively. Hip measurements could not be performed due to the lack of human reference data on this joint.

## 4. Discussion

Experiments showed that the theoretical model of the actuator provides a good approximation of it's output torque and stiffness (Figures 7A,B), which suggests that the actuator can be controlled using a real-time model-based approach instead of relying on load cells, as done by most torque-controlled humanoids, with also direct advantages on mechanical complexity. The frequency response of the driving and variable stiffness mechanisms showed an average bandwidth of 6 and 2 Hz respectively (Figures 8, 9). In practical terms, the system can go from an initial output stiffness of 0 to 5 Nm/deg in about 0.5 s in the absence of external perturbations. It is important to note that these results strongly depend on the used controller and that the main purpose of the tests was to show the ability of the system to change it's frequency response as a function of the stiffness settings. Other control strategies are currently being explored in order to achieve a better performance. However, with the current control strategy, the actuator could effectively modulate it's output position, torque and stiffness in the same range, amplitude and frequency as human joints during walking based on our simulations, as it can be concluded from the small tracking errors observed in Figure 10. The actuator could generate a maximum torque of 40 Nm, with peak to peak values of 65 Nm at the specified gait frequency.

This, in combination with the high position, torque and stiffness control accuracy, suggests the actuator could perform the desired function when implemented in the robot. However, in the current state of the research, these results not yet allow for a conclusive assessment of the biped's performance given the tracking accuracy of the actuator, and other additional higher level control strategies might be required to compensate for the existence of tracking errors.

As presented in section 2.5, the current design only allows to implement 2 compliant actuators (2 VSAs, 2 SEAs or 1 VSA and 1 SEA) into one centralized joint using a cardan. If a third DoF is required, for instance, it becomes necessary to cascade a third actuator connected to the 2-DoF module (see Figure 6), as it's done in the case of the hip and the waist of Binocchio (see Figure 1A). However, based on an analysis done in a previous study from the same authors providing an overview of the key principles of human bipedal walking (Torricelli et al., 2016), we believe it is fair to say that the maximum required amount of DoFs per joint is limited to 3 in order to achieve human-like biped locomotion, including walking under unperturbed and perturbed conditions, and mostly at the hip and waist level. Finally, due to the lack of data for the human waist, we assumed that the same stiffness range as for the rest of the joints applied, which is not necessary correct. However, from the authors point of view, this is a question related to the design choices of the biped robot itself, and not on the design and performance of the VSA, which is the main focus of this study. Therefore, in the authors opinion, the answers to these questions are out of the scope of this paper, and will have to be further investigated in a future study.

Currently, compliance in humanoid robots is mostly used with the goal of improving torque control and/or ensuring safety to shocks, rather than truly replicating human-like joint functions. Our results show that the presented VSA can span from a rigid configuration, up to 5 Nm/deg, to values very close to zero, resulting in a passive behavior of the joint useful when the limb should move freely under inertial or gravitational effects, e.g., during the swing phase. Besides, the actuator presents a stiffening effect with respect to the deviation angle, which has been found beneficial for locomotion (Seyfarth et al., 2006; Vanderborght et al., 2011). Finally, in a previous study the authors demonstrated the ability of the proposed VSA to reduce it's electrical energy consumption online during the execution of repetitive tasks (Jimenez-Fabian et al., 2018). These results suggest that the presented actuator could also reduce the energy requirements of the Biped for walking due to the repetitive nature of this task, which would highly reduce it's cost of transport.

## 5. Conclusions

The understanding of human locomotion has led to usage of VSAs in humanoids due to their inherent advantages. Striving toward a more accurate biologically inspired robotic counterpart of humans, these VSAs have to be implemented in mechanical multi-DoF joints to replicate the rich variety of movements human present. Furthermore, distribution of the extra mass that these VSAs bring forth, due to the required additional motors, is paramount for minimizing the cost of transport of humanoids. However, research toward multi-DoF VSAs is still scarce. This work presented and tested a novel VSA concept designed to be implemented in the sagittal DoFs of the legs of a bio-inspired humanoid robot designed as platform for the validation of biomimetic controllers and the understanding of the neuromechanical processes of human movement, including the role of compliance during walking. The presented actuator allows to place the motors of the VSA in-line with the actuated link and house both motors on the same side of the actuated joint. This not only places the actuator's mass to more proximal locations to create a more favorable mass distribution in the design of the humanoid's leg, but also facilitates it's usage in multi-DoFs joints. The construction of the VSA module, it's main mechanisms, and overall working principle have been explained. The equations necessary to determine it's characteristics have been derived and experimentally validated. The experimental results confirm the functionalities expected from the proposed concept and the accuracy of it's mathematical description. These innovations have been finaly illustrated with the implementation of the VSA module in the multi-DoF joints of the biped Binocchio.

Under a neuroscientific perspective, Binocchio represents a biorobotic test bench that may serve in the future to understand the biomechanical mechanisms of walking performance. The replication of biological joint stiffness dynamics is an emerging and largely unexplored issue that may produce significant step changes in robots operating in real-life environments. This has important implications in robotics, enabling to experimentally validate the hypothesis that stiffness modulation is a determinant for robust and efficient walking, providing invaluable understanding of the neuro-mechanical processes of human movement. Binocchio represents an advanced mechatronic platform that can allow new biologically-motivated walking and standing control algorithms to be directly validated in real-life environment. Besides, beyond humanoid applications, our work has also potential impact in the field of rehabilitation and assistive robotics, where the role of variable compliance has been recently identified as a key factor for the achievement of truly human-like behavior.

## Author Contributions

DR-C, MW, MS-V, and DL conceptualization. DR-C, MW, RJ-F, JG-V, and DT methodology. DR-C and RJ-F software. DR-C and RJ-F validation. DR-C formal analysis. DT, BV, JP, and DL resources. DR-C and MW writing original draft preparation. DR-C, MW, RJ-F, DT, JG-V, MS-V, MS, KB, BV, JP, and DL writing review and editing. JP and DL funding acquisition.

### Conflict of Interest Statement

JG-V is employed by company Ottobock GmbH, but was not employed at the time when the research was conducted. All other authors declare no competing interests. The reviewer SG declared a shared affiliation, though no other collaboration, with one of the authors MS to the handling Editor.
